# The Impact of WhatsApp as a Health Education Tool in Albinism: Interventional Study

**DOI:** 10.2196/49950

**Published:** 2023-11-21

**Authors:** Chetanna Chioma Anaje, Chibuzo Ifeanyi Okpala, Nkechi Anne Enechukwu, Ogochukwu Ifeanyi Ezejiofor, Divinefavour Echezona Malachy, Obumneme Kenechukwu Nwiyi

**Affiliations:** 1 Dermatology Unit Department of Medicine Nnamdi Azikiwe University Teaching Hospital Nnewi Nigeria; 2 Faculty of Medicine College of Health Sciences Nnamdi Azikiwe University Nnewi Nigeria; 3 Department of Mental Health Nnamdi Azikiwe University Teaching Hospital Nnewi Nigeria

**Keywords:** WhatsApp, oculocutaneous albinism, health education, smartphone, mobile phone, teledermatology, photodermatoses, digital health intervention, photoprotection, West Africa, social media, albinism, albino, skin, dermatology, melanin, patient education, sun, sunscreen, discussion group, digital health education

## Abstract

**Background:**

Oculocutaneous albinism is a congenital disorder that causes hypopigmentation of the skin, hair, and eyes due to a lack of melanin. People with albinism are at increased risk of developing skin complications, such as solar keratosis and skin cancers, leading to higher morbidity. As education is crucial in managing albinism, leveraging information technology, such as WhatsApp, can provide an effective intervention for digital health education.

**Objective:**

This study aims to assess the impact of WhatsApp as a tool for providing health education among people with albinism.

**Methods:**

The design of the study was interventional. The intervention consisted of weekly health education sessions conducted in a WhatsApp group for the duration of 4 weeks. The topics discussed were knowledge of albinism, sun protection practices, the use of sunscreen, and myths about albinism. They were all covered in 4 WhatsApp sessions held in 4 separate days. A web-based questionnaire was filled out before and after the intervention by the participants. Mann-Whitney *U* test was used to compare the pre- and postknowledge scores. Spearman correlation was used to correlate data.

**Results:**

The mean age of the study participants was 28.28 (SD 11.57) years. The number of participants was 140 in the preintervention period and 66 in the postintervention period. A statistically significant increase in overall knowledge (*P*=.01), knowledge of sunscreen (*P*=.01), and knowledge of sun protection (*P*<.01) was observed following the intervention. Before the intervention, a positive correlation was observed between age (*r*=0.17; *P*=.03) and education level (*r*=0.19; *P*=.02) with participants’ overall knowledge. However, after the intervention, there was no significant correlation between knowledge and age or education level. A percentage increase of 5.23% was observed in the overall knowledge scores following the intervention.

**Conclusions:**

WhatsApp is an effective tool for educating people with albinism and can act as an alternative to the conventional methods of health education. It shows promising outcomes irrespective of the health literacy level of people with albinism. This educational intervention can positively impact behavior change and translate to consistent sun protection practices. The limitations of this study include the possibility of social desirability bias and data security.

## Introduction

Oculocutaneous albinism is an inherited disorder of hypopigmentation in which there is little or no production of melanin. People with albinism usually have light-colored skin, white hair, and red eyes. Melanin plays a major role in photoprotection and its lack in people with albinism increases the risk of photodermatoses. These may range from benign conditions like freckles or solar lentigos to more severe skin issues, such as nonmelanoma skin cancers—basal cell cancers and squamous cell cancers. Notably, the prevalence of nonmelanoma skin cancers is 20.9% in Nigeria [1], 11.8% in Togo [2], and 26% in Brazil [3]. These sun-related skin conditions increase morbidity and affect the quality of life of people with albinism.

Preventing these photodermatoses includes promoting sun protection practices and educating people with albinism, which is crucial for this purpose. Studies conducted in Africa have documented that health education programs can improve compliance with sun protection practices among individuals with albinism [4,5].

In recent times, IT has revolutionized the dissemination of information, and the use of social media is a prime example. Social media refers to internet-based platforms or application software that enables individuals to communicate, gather, and exchange information, ideas, and images in real time with other users [6]. These platforms, such as WhatsApp, Facebook, Twitter, YouTube, and Instagram, may serve as delivery vehicles for digital health interventions. Social media provides opportunities for public health promotion, patient education, and professional interactions [7]. In addition, it can increase patient engagement and empowerment resulting in better outcomes [8]. On social media, patients with similar conditions and experiences can connect, share knowledge, and support one another [9].

Among the apps employed in digital health education interventions, WhatsApp holds a unique position. It is a messenger app that enables users to make voice calls and send instant messages, photos, videos, and voice messages over the internet [10]. In contrast to the original text messaging function on mobile phones, WhatsApp allows users to send and receive messages at no cost per message [11]. It supports the formation of group chats and permits numerous users to participate, observe, and respond to conversations [10]. It is the third most popular social networking platform in the world, after Facebook and YouTube (which comes second) [12,13]. In Nigeria, WhatsApp is the most preferred social media platform among active users aged 16 to 64 years [14].

Although several studies have used WhatsApp as a health education intervention, there is limited literature available on its application in dermatology in West Africa, specifically for people with albinism [15-17]. The aim of this study was to assess the effectiveness of WhatsApp in health education for this population.

## Methods

### Ethical Considerations

Ethical clearance was obtained from the ethical review board of Nnamdi Azikiwe University Teaching Hospital, Nnewi, Anambra State, Nigeria (NAUTH/CS/66/VOL.16/VER.3/86/2023/039). All study participants gave informed consent. Data anonymity and confidentiality were guaranteed.

### Participants

The study was designed as an educational interventional study. A WhatsApp group was created for the purpose of this study, and it was named People With Albinism Health Education Group. Participants were recruited from various WhatsApp groups for people with albinism in Nigeria, and this was done by sending a group invite link via the group admins to these groups. Participant Disclosure Form (Multimedia Appendix 1) was posted in the People With Albinism Health Education Group. This group had 4 admins who were well-trained dermatologists. Their function was to moderate the conversations in the group and answer questions. A convenience sampling method was used. The inclusion criteria were the following: people with albinism aged 18 years and older or caregivers of children with albinism younger than 18 years of age, who had access to smartphones or tablets and could use WhatsApp.

### Intervention

The WhatsApp messenger app was used as the intervention tool. The participants used their smartphones or tablets. The intervention included weekly sessions of health education delivered through text, images, videos, and voice messages, lasting for a month (January 9 to January 30, 2023). The topics covered in the sessions were the following: (1) understanding albinism, (2) skin problems associated with albinism, (3) sun avoidance and sun protection practices in albinism, as well as (4) myths about albinism and debunking them. In each session, a group admin educated the group for 1 hour (Multimedia Appendix 2). After each session, there was time for questions and comments.

A web-based questionnaire was created using Google Forms, and the link for the form was posted in the People With Albinism Health Education Group. The participants filled out the forms before and after the intervention. The questionnaire consisted of sections consisting of questions on social demographics and 4 domains (ie, questions on understanding albinism, sun protection, the use of sunscreens, and myths about albinism).

The reliability of the questionnaire was analyzed using the Cronbach alpha test and achieved a score of .72. This indicates that the questionnaire demonstrates a reasonable level of internal consistency. The questionnaire was reported in accordance with the Checklist for Reporting Results of Internet E-Surveys (CHERRIES).

### Data Analysis

Data from the web-based questionnaires was downloaded as a CSV file, and data cleaning was done using Microsoft Office Excel (version 2021). Duplicated and incomplete data were removed. Further analysis of data was done using SPSS (version 25; IBM Corp). All continuous variables were tested for normality using the Shapiro-Wilk test and found to be nonnormally distributed; thus, median and IQR values were used as measures of central tendency. Age was further classified into 4 groups based on their quartile distribution. All noncontinuous variables were summarized using frequencies and percentages.

To create a measure of respondents’ knowledge, their responses were graded as follows: strongly disagree=0, disagree=1, neutral=2, agree=3, and strongly agree=4. Responses to questions that included negative or false statements were reverse-scored. The sum of scores for each person was obtained in each domain, namely knowledge of albinism, knowledge of sun protection, knowledge of sunscreens, and knowledge of myths. The percentage score for each person was determined by dividing their sum score by the maximum achievable score in the domain and multiplying by 100. The percentage knowledge scores thus generated were treated as continuous variables.

To compare initial and final responses to individual questions, the Fisher exact test was performed on the frequency of each question. Mann-Whitney *U* test was used to compare the percentage knowledge scores before and after the intervention. Furthermore, Spearman correlation was used to correlate percentage knowledge scores before the start of WhatsApp sessions with age and the highest level of education. We also used Spearman correlation to assess the relationship between percentage knowledge scores after sessions and variables such as the number of sessions attended, age, and the highest level of education. Alpha level was set at *P*<.05.

## Results

**Figure 1:**
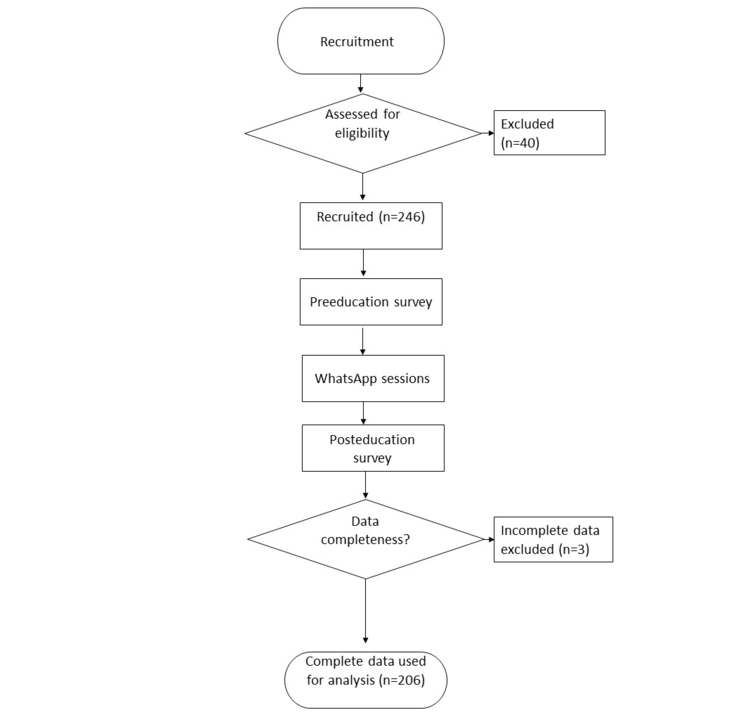
Flowchart of sample selection.

A total of 246 people were recruited in the group. The number of respondents to the web-based survey was 140 before the intervention and 66 after the intervention. Figure 1 shows the flow diagram of the sample selection. Table 1 shows the baseline demographics of the study participants. The median age of the participants was 28 (IQR 22.75-33.25) years. Comparing responses before and after the intervention showed a better understanding of certain knowledge—albinism is inherited, the substance produced in the skin that protects us from the sun is melanin, wide-brimmed hats are better than face caps, and the color of the fabrics is important in sun protection (Multimedia Appendix 3).

**Table 1:** Sociodemographic distribution of participants (N=206).

Characteristics		Frequency, n (%)
**Gender**		
	Male	78 (37.9)
	Female	128 (62.1)
**Are you a person with albinism?**		
	Yes	177 (85.9)
	No	29 (14.1)
**Ethnic group**		
	Igbo	91 (44.2)
	Hausa	16 (7.8)
	Yoruba	67 (32.5)
	Others	32 (15.5)
**Age range (years)**		
	First quartile (≤23)	61 (29.6)
	Second quartile (24-28)	46 (22.3)
	Third quartile (29-35)	48 (23.3)
	Fourth quartile (>35)	51 (24.8)
**Marital status**		
	Single	136 (66)
	Married	68 (33)
	Widowed	2 (1)
**Education**		
	None	1 (0.5)
	Primary	8 (3.9)
	Secondary	44 (21.4)
	Tertiary	153 (74.3)
**Occupation**		
	Unemployed or students	104 (50.5)
	Petty trader, laborer, or messenger	23 (11.2)
	Junior civil servant or senior school teacher	26 (12.6)
	Junior school teacher or artisan	14 (6.8)
	Professional, senior civil servant, or contractor	39 (18.9)
**Have you seen a dermatologist?**		
	Yes	77 (37.4)
	No	129 (62.6)
**Time of filling this questionnaire**		
	Before intervention	140 (68)
	After intervention	66 (32)

Table 2 displays the comparison of the median overall knowledge scores of the study participants before and after the intervention. There was a statistically significant improvement in the overall knowledge scores of the participants after the intervention, particularly in sun protection and sunscreen knowledge. In addition, we found a 5.3% rise in the overall knowledge scores following the intervention. The correlation between age, level of education, number of sessions attended, and knowledge levels was analyzed and presented in Multimedia Appendix 4. There was a positive correlation between age and preintervention overall knowledge scores. Similarly, a positive correlation was seen between the level of education and preintervention overall knowledge scores.

**Table 2:** Comparison of overall knowledge scores before and after sessions. Median and IQR values are presented in percentages.

Characteristics	Preintervention, median (IQR)	Postintervention, median (IQR)	Percentage increase	*U* value	*P* value
Overall score	78.4 (68.6-84.3)	82.5 (69.4-82.4)	5.23	3587	.01
Knowledge of albinism	77.1 (69.4-85.4)	80.2 (68.7-88.0)	4.02	4091.5	.19
Knowledge of sun protection	77.3 (68.1-86.3)	84.1 (72.7-89.2)	8.80	3507	.01
Knowledge of sunscreens	66.7 (55.0-77.7)	72.2 (61.1-83.3)	8.25	3480	<.001
Myths about albinism	91.7 (75.0-95.8)	91.7 (75.0-100)	0.00	4328	.46

Conversely, after the intervention, the correlation of knowledge score with age or level of education was weak and not statistically significant. Moreover, the overall knowledge scores and various knowledge domains correlated significantly with the number of sessions attended. Table 3 shows the normality of all continuous variables using the Shapiro-Wilk test.

**Table 3:** Test for normality of variables.

Variables	Tests of normality (Shapiro-Wilk)	
	Statistic	*P* value
Age	0.980	<.01
Overall score	0.962	<.01
Knowledge of albinism	0.953	<.01
Knowledge of sun protection	0.956	<.01
Knowledge of sunscreens	0.969	<.01
Myths about albinism	0.885	<.01

## Discussion

### Principal Findings

The main finding of this study was a notable improvement in the overall knowledge of participants following the intervention. This study assessed the effectiveness of WhatsApp as a channel for providing health education to people with albinism. It is one of the most popular social media apps in Nigeria and can be used to share information, thereby strengthening the link between health care and IT.

The median age of the participants was 28 years. This is consistent with the age group that uses WhatsApp the most in Nigeria [14]. A notable finding was that 129 (62.6%) of the 206 participants never visited a dermatologist for skin checks. Possible explanations for this may be the paucity of dermatologists in Nigeria and the lack of awareness of the importance of skin checks in albinism. In our intervention, we emphasized the importance of skin checks and encouraged participants to visit dermatologists regularly for such checks.

Significant improvement was seen in the overall knowledge score after the intervention, specifically in the domains of sun protection and sunscreen use. This may be a result of using a combination of texts, images, videos, and audio messages during the intervention. The possibility of using multimedia through WhatsApp distinguishes this app from other digital health interventions that rely solely on text messaging. Mayer [18] stated that well-developed multimedia content can improve learning compared to using a single modality in the educational process [18]. Another reason for the remarkable improvement in overall knowledge after the intervention is the possibility of interaction between the group admins and the participants. This is also enhanced by the fact that WhatsApp group notifications can be received immediately after they are sent. The use of social media platforms, such as WhatsApp, has created room for connecting, sharing, and feedback, thereby enabling better communication [19]. Learning activities carried out through WhatsApp can be effective and impactful, and when used creatively, can serve as an alternative to the face-to-face approach [20].

Comparing the outcomes of this study is difficult due to the paucity of research that used WhatsApp as an intervention tool for health education among individuals with albinism. Pereira et al [15] conducted a pre-post intervention aimed at enhancing breast cancer knowledge among 35 women using WhatsApp, and they reported a statistically significant difference in the postintervention knowledge level. Likewise, Nayak et al [16] and Sartori et al [17] conducted interventional research using WhatsApp to improve knowledge of oral cancer and medication adherence among patients with hypertension and diabetes, respectively. These studies included control groups that received the traditional education method, yet they observed significant differences in the WhatsApp intervention group. Conversely, Al-ak’hali et al [21] indicated that WhatsApp is equally effective as traditional means and reported that WhatsApp had no added benefit compared to traditional education. This could be because, in their study design, all participants in the intervention and control groups had a baseline visit, when they were all educated in the clinic.

Age and educational level of people with albinism correlated positively with the overall knowledge level in the preintervention stage. After the intervention, there was no correlation observed, as the intervention benefitted people with albinism of all age groups and different educational statuses. This implies that the majority of the participants were brought to the same knowledge level irrespective of their age or educational status. This study also demonstrated that the number of sessions attended affected the knowledge levels, highlighting the importance of early involvement of the participants in such interventions to achieve maximum impact. Overall. These findings showed that WhatsApp is a valuable tool in digital health interventions that can be explored in the field of dermatology.

The increased knowledge among participants following our intervention may lead to behavior-based sun protection practices. This adjustment in behavior can be reinforced by periodic conversations and reminders regarding albinism and photoprotection in the WhatsApp group. Recent reviews of social media interventions for health behavior change have shown that social media can have a substantial effect on health behavior [22].

With regard to myths of albinism, we noted that there was no statistical significance in knowledge levels before and after the intervention. A possible explanation for this is that most of the participants had a good understanding of the myths about albinism before the intervention. This can be attributed to the educational status of the participants. The highest educational level of most of the participants was tertiary education, indicating that they were sufficiently informed to distinguish between myths and facts about albinism. This is supported by the finding that knowledge of myths was significantly correlated with the educational status of respondents.

Further work will be required to explore the long-term impact of WhatsApp-based health education on sun protection practices among people with albinism and to investigate the scalability and cost-effectiveness of such interventions in larger populations.

### Limitations

As an interventional study, there is a possibility of social desirability bias [17]. This can be seen in health interventions for individuals with chronic diseases [23]. Participants would be likely to portray themselves or certain behaviors in a favorable light.

Data security is another limitation of WhatsApp. Although WhatsApp offers end-to-end encryption of messages that ensure the security of information, there is still the chance of data security breaches.

Additionally, the smaller number of postintervention participants compared to the number of participants before the intervention probably affected the overall power of the study.

### Recommendations

Further studies could be conducted on the long-term effectiveness of WhatsApp in attitudes and behavior changes related to photoprotection in people with albinism. Additionally, research with a control group of people with albinism undergoing traditional methods of communication could allow for a comparison between WhatsApp and traditional means of health education. Further research on expanding the use of mobile apps in dermatology would improve health care services, especially in Nigeria.

### Conclusions

WhatsApp has been useful in the health education of people with albinism, providing them with knowledge on albinism, sun protection practices, and myths about albinism. Interactive messaging and the use of multimedia were important in the success of this intervention program. Therefore, WhatsApp has proven to be a promising tool in teledermatology in Nigeria. There is a paucity of studies evaluating the effectiveness of using WhatsApp in the health education of people with albinism. However, we consider this study to be the first of its kind in demonstrating it as a suitable approach.

## Appendix

Multimedia Appendix 1 Participant disclosure form.

Multimedia Appendix 2 Screenshots of the intervention.

Multimedia Appendix 3 Comparison of responses to questions before and after WhatsApp sessions.

Multimedia Appendix 4 Correlation of age, highest level of education, and the number of sessions attended with knowledge levels.

## Abbreviations

CHERRIES: Checklist for Reporting Results of Internet E-Surveys
