# Supplementary material for: The Impact of WhatsApp as a Health Education Tool in Albinism: Interventional Study
Source: JMIR Dermatol. 2023 Nov 21;6:e49950. doi: 10.2196/49950 (PMC10698648; doi:10.2196/49950)
Supplement: Multimedia Appendix 1 [file derma_v6i1e49950_app1.docx]

Multimedia Appendix 1

**PARTICIPANT DISCLOSURE FORM**

**Study Title**: The Impact of WhatsApp as a Health Education tool in Albinism

**Introduction**: We invite you to participate in our study aimed at assessing the impact of WhatsApp as a tool for providing health education to persons with albinism (PWAs). Before you proceed, please carefully read the following information regarding the questionnaire and data storage:

**Survey Length and Time Commitment**:

- The survey consists of questions about how much you know about albinism, sun protection practices, use of sunscreen, and myths associated with albinism.
- It should take between 7 to 10 minutes to fill out the questionnaire.
- We appreciate your time and your effort to take part in this questionnaire.

**Data Storage and Privacy**:

- Your responses will be collected using Google Forms.
- All data collected will be kept strictly confidential and used solely for research.
- No personally identifiable information will be linked to your questionnaire responses.
- Data will be stored securely on password-protected computers and may be accessible only to the research team.
- Data will be retained for 3 years in accordance with research data retention policies.
- Your participation in this study is voluntary, and you may withdraw at any time without penalty.

**Benefits and Risks**:

- Your participation will contribute to advancing knowledge about the effectiveness of WhatsApp-based health education.
- There are no known risks associated with participating in this survey.

**Contact Information**:

- If you have any questions or concerns about the survey or your participation, please contact any of the group admins of the PWA WhatsApp groups.

**Consent**:

- By proceeding with this questionnaire, you acknowledge that you have read and understood the information provided in this disclosure form.
- Your decision to participate in this questionnaire is entirely voluntary, and you may choose to exit the survey at any time without penalty.
- If you agree to participate, please continue to the questionnaire by clicking the link below this form.

Thank you for considering participation in our study. Your contribution is greatly appreciated.

Top of Form
