# Supplementary material for: The Impact of WhatsApp as a Health Education Tool in Albinism: Interventional Study
Source: JMIR Dermatol. 2023 Nov 21;6:e49950. doi: 10.2196/49950 (PMC10698648; doi:10.2196/49950)
Supplement: Multimedia Appendix 2 [file derma_v6i1e49950_app2.pdf]

14:16 42%

PWA Health education grp
only admins can send messages

Less than 1 hour to go!

Outline of the discussions for Jan 2023

9th Jan - Understanding Albinism
16th Jan - Skin problems seen in albinism
23rd Jan - Sun protection and sun avoidance practices
30th Jan - Myths about albinism - debunking them!
14:14 ✓
❤️👍 3

YOU ARE WELCOME TO THE FIRST HEALTH EDUCATION SESSION!
15:03 ✓
❤️🔥 7

Hello!👋🏽👋🏽

Good afternoon to everyone!😀

It is so good to have you all and you are highly welcome to our first weekly session of health education.
15:05 ✓
❤️👍 10

I am Dr Chetanna Anaje, a consultant dermatologist (skin doctor) and I work in Nnamdi Azikiwe University Teaching Hospital, Nnewi, Anambra State.
15:05 ✓
❤️🙏👍 12

In my hospital, persons with albinism (PWAs) hold a special place in our heart as we organize various activities to keep sensitizing and spreading the knowledge about albinism and being sun-smart.☀️☀️☀️
15:06 ✓
❤️👍 10

Before we talk about albinism, we will talk about the skin.
15:07 ✓
❤️👍 4

The skin is the outermost layer of our body and serves different functions such as controlling our body temperature, helps in the production of Vitamin D and other functions.
15:07 ✓
👍😮 4

Another function of the skin is to protect us from the harsh effects of the sun. ☀️☀️The way it does that is by a substance called **Melanin.**
15:08 ✓
👍 3

Melanin is made in the skin and it gives color to the skin, eyes 👀and hair. It also absorbs the harsh sun rays☀️☀️☀️ that hit our skin.
15:10 ✓
👍❤️ 6

If these sun rays are not absorbed by melanin, it will cause problem to the skin such as sun burns, freckles and so on.

It can also cause serious problems such as skin cancers.
15:11 ✓
❤️😮👍 5

In albinism, it is an **INHERITED** condition in which the skin is producing **little or no melanin.**
15:12

Message

It can be seen in **ALL races** and **ethnic groups.** So, it is not only in Nigeria 🇳🇬, even in Tanzania 🇹🇿 America 🇺🇸, Brazil 🇧🇷, India 🇮🇳 ,etc.
15:16

❤️ 👍 7

It affects BOTH males 👨🏼 and females 👩🏼
15:17

❤️ 👍 5

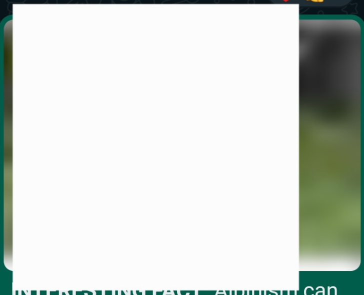

INTERESTING FACT: Albinism can occur in plants and animals too! The mother deer is white while the offspring is brown .
15:22

👍 ❤️ 😮 7

There are **2** forms of Albinism: Oculocutaneous (OCA) and Ocular (OA).

OCULOCUTANEOUS ALBINISM is when there is little or no melanin in the skin, hair and eyes.

OCULAR ALBINISM is when there is little or no melanin in the eyes alone.
15:26

😮 5

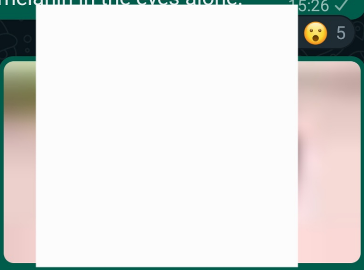

Ocular albinism - Note that the eyes are blue. For some, it may another color.
15:27

👍 ❤️ 5

The cause of albinism is a **problem** in one of several **genes** that produce or distribute melanin, the pigment that gives skin, eyes 👁️, and hair their coloring.

The problem may result in the absence of melanin production or a little amount of melanin production.
15:29

👍 ❤️ 😮 8

For most types of albinism, both parents 👫 must carry the gene in order for their child to develop the condition.

Most people with albinism have parents 👫 who are only carriers of the gene and don't have symptoms of the condition.
15:31

❤️ 👍 😮 5

Due to the lack of melanin in the skin of PWA, the skin is lighter and more sensiti[illegible]

Message

Due to the lack of melanin in the skin of PWA, the skin is lighter and more sensitive to the sun.

The skin does NOT tan – it means it cannot get darker or browner on sun ☀️ ☀️exposure.

The skin is not only affected, the eyes 👁️ are also affected for some too – problems with vision.

15:33 ✓

❤️ 👍 😮 6

There is **NO CURE** for albinism but several steps are taken to reduce or prevent the harsh effect of the sun ☀️☀️.

Our people talk say prevention is better than cure or according to my Waffi people, *provision store dey better than kiosk!* 😁 😁

(There is a session that will hold on the 23rd Jan to discuss sun ☀️protection)

15:37 ✓

😂 ❤️ 👍 🥰 8

NEXT WEEK 📅📅 ,we will talk about different skin problems that can occur in albinism.

15:39

👍 ❤️

Message

14:18 41%

PWA Health education grp
only admins can send messages

Good afternoon all!👋🏽

It is about 1 hour to go!

Topic for today: **Skin problems seen in Albinism**

After the session, I will enable our dear participants to type so that questions can be asked and comments taken.

See you soon! 🙂🙂

13:59 ✓

❤️👍 13

Good afternoon everyone once again!👋🏽👋🏽

You are all welcome to the 2nd 2️⃣ session of this Health education series!

Our topic is **Skin problems seen in Albinism**

15:04 ✓

❤️👍🏼👂🏼 7

My name is Dr Chetanna Anaje, a consultant dermatologist at Nnamdi Azikiwe University Teaching Hospital, Nnewi, Anambra State.

15:05 ✓

👍❤️ 3

We thank those that sent their questions and comments. 🙏🏽🙏🏽🙏🏽

We are really blown away by the feedback. 😊😊

15:06 ✓

❤️👍 2

As a way of recap, we will remind ourselves of what albinism is.

📍 Albinism is a skin condition in which there is little or no melanin production.

📍📍 Melanin is the substance that protects us from the harsh effects of the sun.

📍📍📍 Due to the little or no melanin in the skin, there is increased sensitivity to the effects of the sun ☀️☀️

📍📍📍📍 Sun ☀️ has very harsh rays known as ultraviolet rays or you can call it UV rays. These UV rays can cause problems in the skin especially in the skin of PWA.

15:10 ✓

❤️👍 8

These sun rays🌞 can cause various skin problems such as Sunburns and others.

We will look at them one by one

15:13 ✓

😮

A. SUNBURNS

Sunburns can occur from excessive sun 🌞 exposure.

There may be redness of the skin 🥵 with blisters. Sometimes, itching, stinging or

Message

14:19 41%

PWA Health education grp
only admins can send messages

A. SUNBURNS

Sunburns can occur from excessive sun 🌞 exposure.
There may be redness of the skin 🥵 with blisters. Sometimes, itching, stinging or burning sensation 🔥 may occur.
15:17 ✓

👍😂❤️😮 5

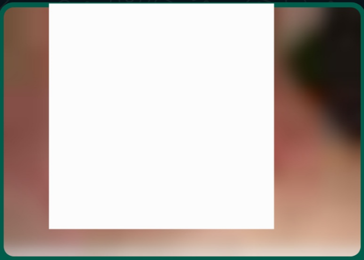

Example of sunburn. This one is a severe type of sunburn.
15:21 ✓

😮👍😢 4

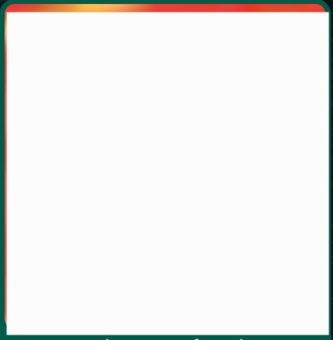

How to take care of sunburn
15:24 ✓

❤️👍 6

Taking care of Sunburns

1.Take frequent cool baths 🛁 or showers 🚿 to help relieve the pain. As soon as you get out of the bathtub or shower, gently pat yourself dry, but leave a little water on your skin. Then, apply a moisturizer to help trap the water in your skin. This can help ease the dryness.

2. Use a moisturizer that contains aloe vera to help soothe sunburned skin.

3. Consider taking pain killers like Paracetamol or Ibuprofen to help reduce any swelling, redness and discomfort 🤢.

4. Drink extra water 💧.

5. If your skin blisters, allow the blisters to heal.

6. You should not pop the blisters, as blisters form to help your skin heal and protect you from infection.

7. Take extra care to protect sunburned skin while it heals. Wear clothing 👖 ...
Read more
15:32 ✓

❤️👍😢 8

B. FRECKLES/ MOLES / LENTIGOS

Freckles are small brown spots on your

Message

14:19 41%

PWA Health education grp
only admins can send messages

B. FRECKLES/ MOLES / LENTIGOS

Freckles are small brown spots on your skin. Unlike moles, which are raised, freckles are flat. Freckles aren't painful or harmful.

Solar lentigo or lentigines are harmless patch which is dark in color. They are not painful either.
15:38 ✓

👍❤️😮 6

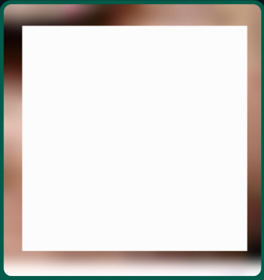

Lentigo or solar lentigo
15:40 ✓

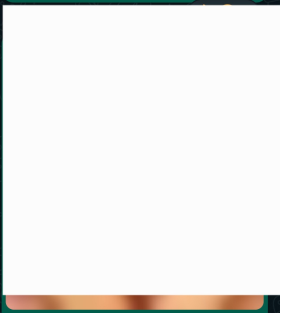

Freckles.
15:43 ✓

😮

Freckles and lentigoes are not cancers but they may be confused with skin cancer.

➡️ If you notice changes in size, color, or shape of a freckle, see your doctor or dermatologist. They'll be able to determine whether it's a reason for concern.
15:45 ✓

👍 3

Treatment of Freckles and lentigoes

Sunscreens are important

Treatment options that may be recommended by your dermatologist may include
* topical creams
** chemical peels
*** laser, etc.
15:48 ✓

👍❤️ 2

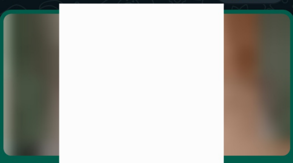

C. ELASTOSES OR SOLAR ELASTOSES

The skin can become thick and have a yellowish tinge to it.
15:54 ✓

👍😲😮 3

Treatment of elastosis
Meet your skin doctor who may prescribe for you* topical cream
** Chemical peels
*** Laser treatment
15:56 ✓

👍 2

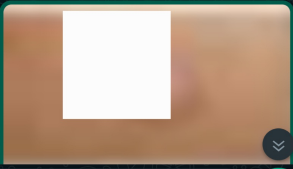

Message

D. ACTINIC KERATOSES

These are patches that may be dry and have scales.

It may look like small wounds that does not want to heal.

Please when you notice these little small wounds, try and see a skin doctor 🥼🥼.

This is because solar keratosis can become skin cancer 🥴 . 15:59

😮🏃👍 4

E. SKIN CANCERS

Different skin cancers can occur from long sun 🌞 exposure. There is one called basal cell cancer (BCC) and other called squamous cell cancer (SCC).

Basal cell cancer can appear as ulcers or wounds with rolled edges. The edges can be like doughnut 🍩.

Squamous cell cancer (SCC) of the skin – It can start from actinic keratosis. Appears like a wound that never heals.

*SCC is like actinic keratosis wey dey University or Polytechnic. 🙂 16:03

👍✍️😢 5

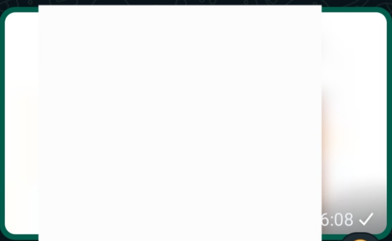

[illegible]6:08

😮

The basal cell cancers and squamous cell cancers **can be cured** if caught early and may not cause too much problems at the early stage.

When squamous cell cancer is found late, it may be challenging or difficult to treat.

Basal cell cancer does not cause as much problem as the squamous cell cancer if found late. It will be affecting the normal skin surrounding it very slowly 🐌.

The treatment of these cancers is to remove that cancer tissue which can be done through surgery. 16:14

👍😢😮 5

** Apart from the skin problems mentioned above, some PWAs may have birthmarks. These are harmless and not cause for alarm. 16:16

❤️👍 2

The importance of these session is to have an idea of the skin problems that ca[illegible] occur and to **VISIT THE DERMATOLOGIS[illegible]**

Message

It may be challenging or difficult to treat.

Basal cell cancer does not cause as much problem as the squamous cell cancer if found late. It will be affecting the normal skin surrounding it very slowly 🐌.

The treatment of these cancers is to remove that cancer tissue which can be done through surgery. 16:14

👍 😢 😮 5

** Apart from the skin problems mentioned above, some PWAs may have birthmarks. These are harmless and not cause for alarm. 16:16

❤️ 👍 2

The importance of these session is to have an idea of the skin problems that can occur and to **VISIT THE DERMATOLOGIST or SKIN DOCTOR** 🥼 🩺 **EVERY 6 MONTHS OR 12 MONTHS** 📅 16:18

👍 2

You should also visit the skin doctor **WHEN YOU NOTICE A STRANGE RASH ON YOUR SKIN!!!** 16:19

👍 🙏 3

THANK YOU!
Daalu! Na gode! É seun! Anya! M sugh u! Sosono eti eti! Agba! Usa! 16:25

👍 ❤️ 🙏

14:21 41%

PWA Health education grp
only admins can send messages

Good afternoon, everyone, and it's very nice to be here 15:08
🙏🏾 👍 4

I am Dr Chibuzo Okpala, as has been introduced 15:09
👍 ❤️ 4

My job has been made easier by the previous talks given by my colleague 15:10
👍 3

The topic today is Myths in Albinism 15:10
👍

Myth is defined as a widely held but false belief or idea 15:11
👍🏽 ❤️ 3

This means that everything we would say here relating to albinism are all false 😂 15:12
😀 😮 2

You have been told the truth about albinism. It's time to hear about some of the false things that fly around about the condition 15:13
👍🏽 ✍🏻 3

Sadly, some af these myths have caused great harm and discrimination against PLWA 15:13
👍

But, thankfully, most of the dangerous practices resulting from some of these myths occur mostly outside of Nigeria 15:15
👍

They are more common in Eastern and Southern Africa 15:15

I will go through some of those myths and would welcome other ones from the audience when the chat room is opened 15:16
👍🏽 3

The following are some of the myths that fly around and the factual statement about them 15:18

Myth 1: Mother of PLWA was impregnated by a white man

Fact: This is not true as we now know albinism is a genetic condition 15:19
👍🏽 😁 ❤️ 5

Myth 2: the Devil replaces the original baby with PWA

Fact: this is not true at all, albinism is a genetic disorder 15:20
👍🏽 🙃 😂 5

Myth 3: Albinism is a contagious condition:

Fact: Albinism is not contagious. It is genetic 15:21
👍🏽 😂 4

Myth 4: PWA have special powers and [illegible]

Message

14:22 41%

PWA Health education grp
only admins can send messages

Myth 4: PWA have special powers and bring good luck and money when their body parts are used as charm. This has sadly led to the killings of lots of PWA in Tanzania, Zimbabwe, and other African countries

Fact: This is a big lie spread by witch doctors in those areas for selfish reasons, PWA are just like any other person and has no such powers,
15:25

👍😂 7

Myth 5: Albinism is due to lack of sun, leading to exposure of PWA to the sun to reverse the condition

Fact: This is not true. In fact, exposing them to the sun would harm their skin, leading to lots of skin lesions, including cancers
15:27

👍🚶😂 6

Myth 6: Ritual defilement and rape of girls with Albinism can cure HIV/AIDs

Fact: This is a very unfortunate lie, which rather leads to unfortunate transmission of HIV/AIDs to those innocent girls
15:30

😢😮😂🙏 10

Myth 7: PWA are sterile (infertile)and can't bear children

Fact: This is another big lie. Albinism does not affect fertility in any way. PWA can give birth to as many children as they wish
15:33

👍🙄❤️ 5

Myth 8: PWA are caused and should be discriminated against

Fact: PWA are blessed and should be given consideration in employment and other aspects of life
15:35

👍❤️ 6

Myth 9: PWA have a short life span

Fact: Albinism does not affect the life span of the individual. They only need to make sure they protect their skin from the sun to prevent complications like squamous cell cancers
15:37

❤️👍 8

Myth 10: PWA are mentally retarded or deaf

Fact: there is no truth in these, PWA have normal mental functions and no hearing deficit
15:39

👍❤️😂😆 7

Myth 11: Albinism results from a mixture of two races

Fact: This is not true as it is seen in people of the same race.
15:41

👍❤️ 6

Myth 12 : PWA are blind

Fact: though, albinism affects sight, it doesn't lead to blindness and the

Message

14:22 41%

PWA Health education grp
only admins can send messages

Myth 12 : PWA are blind

Fact: though, albinism affects sight, it doesn't lead to blindness and the defects can easily be corrected 15:43

👍❤️😮 5

I hope I have been able to go through some of the common myths that fly around, which are all false

The true facts about albinism have been shared in previous talks and in other talks to come

There may be more myths flying around our various localities, and we encourage the audience to share some of those myths when the chat is open for clarification

We are thankful that some of those dangerous myths that lead to harm of PWA are not common in Nigeria, but we would continue to interface with the government and society at large to stop completely all forms of discrimination against PWA

Thank you very much, and enjoy the rest of your day 15:49

❤️🤝👍🙏 9

Thanks a lot for sharing!😀😀 Thanks for busting these myths!🙏 🙏

Pls, the chat room will be opened for people to ask questions and make comments!

After 24 hours, it will be closed.

Let's keep our questions and comments on the topic of our discussion! 15:51 ✓

You changed this group's settings to allow all participants to send messages to this group

14:23 40%

PWA Health education grp
only admins can send messages

Once again, I am Dr Chetanna Anaje, a dermatologist at Nnamdi Azikiwe University Teaching Hospital, Nnewi, Anambra State. 🙂 15:03

We will be talking about 🌞 Sun Protection and sun avoidance practices today. 15:04

As a recap, in albinism, there is little or no production of melanin in the skin.

Melanin is involved in protecting our skin from the harsh effects of the sun 🌞.
In our previous session, we also talked about the skin problems associated with exposure to the sun for a long time which includes lentigines, elastoses, actinic keratosis, skin cancers. Skin cancer is one of the most preventable cancers.

Last week, we debunked several myths of albinism which was very important.
15:06
👍 ❤️ 6

The task of preventing damage to the skin over a lifetime can be challenging. 15:06
👍 ❤️ 5

When you protect your skin from the sun's harmful rays ☀️, you reduce the risk of developing sun damage to skin. 15:08
❤️ 👍 ✍️ 3

I will talk about ways to practice sun safety 15:09
👍 3

📍 First of all, SEEK SHADE. Seek shade when appropriate, remembering that the sun's rays are the strongest between 10 a.m. and 2 p.m. You can also look at your shadow. Any time your shadow appears shorter than you, seek shade.

(Please don't ask me how the sun 🌞 makes the shadow becomes shorter, it is beyond my scope! This is one of the reasons I skipped Geography as a subject in my Senior Secondary!)😂😂 15:11
😂 🤦 🤣 😮 15

📍 📍 Secondly, wear sun-protective clothing 👖 🧥 👘.

Things to consider
Color: Dark-colored clothing is better than lighter shades. Colored clothing allow less light penetration.

Type: All fabrics offer some level of sun 🌞 protection but the examples of fabrics that offer a higher level include polyester, nylon, silk .

Weave: Loosely woven fabrics provide less protection than tightly woven fabrics. To see how tight the weave on a piece of clothing is, hold it up to a light. If you can see light through it, the weave may be too loose to be effective at blocking the sun's rays.

Wetness 💦 💦: Dry fabric provides more protection than wet fabric. Wetting a fabric reduces its effectiveness by as much as 50%. In addition, if you're at the beach or pool, keep in mind th... Read more
15:17
❤️ 👍 😮 5

Other factors to consider
Coverage: The more skin your outfit covers, the better your protection

In developed countries, 🌞 protective clothing is practically designed and uses fabrics that are breathable and light to make it comfortable to wear in warm, sunny conditions. Specific additives are added to the fabric that help in sun protection. They are referred to as UPF(Ultraviolet Protection Factor) and it is

Message

14:24 40%

PWA Health education grp
only admins can send messages

In developed countries, 🌞 protective clothing is practically designed and uses fabrics that are breathable and light to make it comfortable to wear in warm, sunny conditions. Specific additives are added to the fabric that help in sun protection. They are referred to as UPF(Ultraviolet Protection Factor) and it is put in their labels.
15:20 ✓

❤️ 👍 7

Hats 👒👒👒are essential. Hats with brims at least 3 inches wide all the way around are best for protecting the face 👨 👩 ears 👂, and neck. Hats with narrower brims, such as 🧢🎩🎓⛑️, may not give sufficient sun protection.

A hat 👒👒is a simple and effective way to cover up your face and neck. When selecting a hat, choose one that has a wide brim 👒, which will protect your ears 👂, as well as your head and neck.
15:25 ✓

❤️ 👍 5

Shoes 🥿👞👟👞👠 that cover your feet are also important. 👌
15:27 ✓

👍 😮 ❤️ 4

Sunglasses 🕶️ with UV protection are also important.

Sunglasses are an important part of your sun-protective wardrobe.

When purchasing sunglasses, always look for lenses that offer UV protection. Lenses that appear dark do not necessarily offer UV protection, so make sure to read the label before purchasing or make enquiries.

Large-framed or wraparound sunglasses are preferred.😉
15:29 ✓

👍 ❤️ 5

[illegible]

14:24 40%

PWA Health education grp
only admins can send messages

Thirdly, USE SUNSCREENS AND SUNBLOCKS.

Sunscreen is a chemical defense, penetrating the skin and absorbing the UV rays before they reach and damage the deeper parts of the skin.

Sunblock is a physical way to defend against ultraviolet (UV) rays ☀️☀️. It sits on top of the skin and acts as a barrier 🚫 . Typically, sunblock includes zinc oxide or titanium oxide. Sunblocks are often opaque and noticeable when applied to the skin.

Many brands of sun protection offer a blend of sunscreen and sunblock. 15:33

👍 3

What is SPF?
SPF is an acronym for sun protection factor. It's an indication of how well a product will actually protect you from the sun's ultraviolet B (UVB) rays ☀️☀️

The SPF number tells you the amount of time it takes for the skin to redden upon exposure to the sun with protection as opposed to the amount of time without protection.

If used exactly as directed, a product with SPF 30 will take the sun 30 times longer to burn the skin than skin directly exposed without protection. A product with SPF 50 will take 50 times longer.

I hope we are understanding and I am not becoming too technical! 🤔 15:35

👍❤️ 8

THINGS TO NOTE

A. If you have a choice, it's best to choose a sunscreen with broad-spectrum UV protection of at least **SPF 30.**

B. Ideally, you should also apply sunscreen to your skin **30 minutes** before going out into the sun.

C. Sunscreen lasts an average of two 2️⃣ hours. That means you should plan to reapply every two 2️⃣ hours. (I know it is not easy!) 15:38

❤️😮👍 6

Some sunscreens are made commercially and sold. However, a lot of us have complained of the cost. *Some people don talk say na after man find something to eat, then man go apply sunscreen. Everywhere tight* 😊😊

However, I have heard of homemade or DO IT YOURSELF (DIY) sunscreens. Some people add zinc oxide powder to their body lotion or cream.

DISCLAIMER: I HAVE NOT HAD PERSONAL EXPERIENCE WITH IT NOR HAVE I MADE IT MYSELF. Therefore, I AM NOT RESPONSIBLE FOR ANY SIDE EFFECTS CAUSED BY THIS. THERE IS

Message

14:24 • 40%

PWA Health education grp

only admins can send messages

Practicing sun prevention and Avoidance is not easy.

I want to applaud 👏 and give *5 Gbosas* those of us who are practicing it.

For some of us that are not observing it, it is important we do it as much as we can and be consistent.

15:47 ✓

👍 ❤️ 6

Sometimes when people come down with skin problems later as adults, it is as a result of frequent and consistent exposure to the sun uncovered as a child.

As a child, your skin is very beautiful but when we become adults, we start having issues with our skin. Just like it is said, *Rome was not built in a day*.

A little sun ☀️ exposure here and a little sun ☀️☀️ exposure there contribute to sun damage to the skin.

15:50 ✓

👍

Let's keep being sun 🌞 smart😎🤓!

We can live our lives to the fullest by being sun smart and sun safe!

15:51 ✓

👍 ❤️ 5

Thank you all for reading! I appreciate your audience!

15:51 ✓

❤️ 👍 6
