# Supplementary material for: The Impact of WhatsApp as a Health Education Tool in Albinism: Interventional Study
Source: JMIR Dermatol. 2023 Nov 21;6:e49950. doi: 10.2196/49950 (PMC10698648; doi:10.2196/49950)
Supplement: Multimedia Appendix 3 [file derma_v6i1e49950_app3.docx]

Multimedia Appendix 3: Comparison of responses to questions before and after WhatsApp sessions.

|  | SD %  1^st^(2^nd^) | D%  1^st^(2^nd^) | N%  1^st^(2^nd^) | A%  1^st^(2^nd^) | SA%  1^st^(2^nd^) | F(*P*) |
| --- | --- | --- | --- | --- | --- | --- |
| Knowledge of albinism |  |  |  |  |  |  |
| Albinism affects the skin, eyes and hair. | 4.3(6.1) | 2.9(1.5) | 0.7(0) | 37.9(34.8) | 54.3(57.6) | 1.32(.91) |
| Albinism is inherited. | 4.3(4.5) | 11.4(4.5) | 10(1.5) | 40.7(37.9) | 33.6(51.5) | 10.62(.02) |
| The skin plays a small role in protecting us from the harsh sun rays.^a^ | 14.3(13.6) | 19.3(9.1) | 5.7(1.5) | 34.3(39.4) | 26.4(36.4) | 6.35(.16) |
| The substance that is produced in the skin that protects us from the sun is melanin. | 3.6(0) | 2.9(1.5) | 2.9(1.5) | 40(24.2) | 50.7(72.7) | 9.29(.03) |
| In albinism, the skin is producing enough melanin.^a^ | 47.9(57.6) | 27.1(27.3) | 6.4(3) | 12.9(12.1) | 5.7(0) | 5.47(.23) |
| Skin problems can occur in PWA as a result of frequent exposure to sun. | 4.3(3) | 0(0) | 2.1(1.5) | 27.9(28.8) | 65.7(66.7) | 0.30(1.00) |
| Sun burns can occur from frequent sun exposure. | 1.4(0) | 0.7(0) | 1.4(1.5) | 30(36.4) | 66.4(62.1) | 1.93(.84) |
| Brown or black spots can also occur from frequent sun exposure. | 0.7(0) | 2.1(1.5) | 5(3) | 34.3(42.4) | 57.9(53) | 1.88(.82) |
| Thickening of the skin and rough skin can occur from frequent sun exposure. | 0(0) | 3.6(1.5) | 7.1(6.1) | 39.3(36.4) | 49.3(56.1) | 1.01(.82) |
| Actinic keratoses can occur from frequent sun exposure. | 0(0) | 2.9(1.5) | 9.3(4.5) | 46.4(40.9) | 41.4(53) | 3.00(.39) |
| Skin cancers can occur from frequent sun exposure. | 2.1(1.5) | 1.4(0) | 5(1.5) | 31.4(31.8) | 60(65.2) | 2.02(.76) |
|  |  |  |  |  |  |  |
| Knowledge of sun protection |  |  |  |  |  |  |
| The skin of many people with albinism can tan (or become browner). | 10.7(12.1) | 13.6(16.7) | 16.4(9.1) | 39.3(37.9) | 20(24.2) | 2.57(.63) |
| Avoiding the sun and protecting your skin from the sun exposure is important. | 2.1(0) | 0(0) | 0(0) | 29.3(21.2) | 68.6(78.8) | 2.61(.22) |
| Staying away from the sun from 11 am to 2 pm is important. | 1.4(0) | 1.4(0) | 0.7(3) | 37.9(34.8) | 58.6(62.1) | 2.99(.53) |
| Using an umbrella when going out under the sun is necessary. | 0.7(0) | 0(0) | 1.4(1.5) | 32.9(34.8) | 65(63.6) | 0.76(.96) |
| Wearing hats are also important when going out. | 0(0) | 0(0) | 2.1(1.5) | 39.3(36.4) | 58.6(62.1) | 0.31(.90) |
| Face caps are better than wide brimmed hats for sun protection. ^a^ | 20(34.8) | 37.9(42.4) | 12.1(4.5) | 21.4(10.6) | 8.6(7.6) | 9.70(.04) |
| Wearing long sleeved clothing is one way of sun protection. | 1.4(1.5) | 0.7(0) | 1.4(0) | 37.9(39.4) | 58.6(59.1) | 1.26(1.00) |
| The color of the fabric is important when wearing clothes for sun protection. | 0.7(0) | 8.6(1.5) | 9.3(6.1) | 49.3(42.4) | 32.1(50) | 8.57(.04) |
| Dark colored clothing offers better sun protection than light colored clothing. | 20.7(24.2) | 25(19.7) | 15.7(7.6) | 22.9(33.3) | 15.7(15.2) | 4.88(.29) |
| Seeking shades under trees and buildings is one of the ways of sun protection | 0.7(1.5) | 5.7(3) | 8.6(3) | 58.6(53) | 26.4(39.4) | 5.69(.20) |
| Sunglasses are useful in protecting your eyes from the sun. | 0.7(0) | 0.7(0) | 0.7(0) | 47.1(42.4) | 50.7(57.6) | 2.00(.83) |
| Regular visits to the skin doctors for skin inspection is unnecessary. | 30.7(36.4) | 23.6(33.3) | 5(1.5) | 24.3(13.6) | 16.4(15.2) | 5.68(.22) |
|  |  |  |  |  |  |  |
| Knowledge of sunscreens |  |  |  |  |  |  |
| Sunscreens play a small role in sun protection.^a^ | 16.4(19.7) | 27.1(27.3) | 4.3(3) | 37.1(42.4) | 15(7.6) | 2.69(.61) |
| Sunscreens should be applied daily on the skin. | 1.4(1.5) | 2.1(4.5) | 7.9(4.5) | 53.6(53) | 35(36.4) | 1.88(.79) |
| Sunscreen should be reapplied after sweating or swimming. | 2.9(1.5) | 7.1(6.1) | 11.4(6.1) | 51.4(56.1) | 27.1(30.3) | 1.88(.77) |
|  |  |  |  |  |  |  |
| Knowledge of myths surrounding albinism |  |  |  |  |  |  |
| Albinism is contagious. ^a^ | 59.3(65.2) | 22.1(19.7) | 6.4(1.5) | 7.9(9.1) | 4.3(4.5) | 2.64(.63) |
| Albinism is caused by evil spiritual or supernatural powers. ^a^ | 78.6(71.2) | 15(22.7) | 2.9(0) | 0.7(3) | 2.9(3) | 5.10(.23) |
| Persons with albinism have normal intelligence. | 0.7(3) | 3.6(3) | 5.7(3) | 30(34.8) | 60(56.1) | 2.76(.61) |
| Persons with albinism have normal life span | 1.4(1.5) | 3.6(1.5) | 2.9(1.5) | 33.6(34.8) | 58.6(60.6) | 0.97(.95) |
| Eating or adding salt in the food in persons with albinism can cause sunburns. ^a^ | 48.6(51.5) | 33.6(37.9) | 4.3(3) | 10(3) | 3.6(4.5) | 3.46(.48) |
| Persons with albinism can have children. | 2.1(0) | 0.7(0) | 0(0) | 20.7(27.3) | 76.4(72.7) | 2.34(.55) |

**^a^** The responses were scored differently as follows: strongly disagree = 4, disagree = 3, neutral = 2, agree = 1, strongly agree = 0.
