# Supplementary material for: The Impact of WhatsApp as a Health Education Tool in Albinism: Interventional Study
Source: JMIR Dermatol. 2023 Nov 21;6:e49950. doi: 10.2196/49950 (PMC10698648; doi:10.2196/49950)
Supplement: Multimedia Appendix 4 [file derma_v6i1e49950_app4.docx]

Multimedia appendix 4: Correlation of age, highest level of education and number of sessions attended with knowledge levels.

|  |  |  | Overall scores | Knowledge of albinism | Knowledge of sun protection | Knowledge of sunscreens | Myths about albinism |
| --- | --- | --- | --- | --- | --- | --- | --- |
| Age | 1^st^ ^b^ | R(*P*) | 0.17(.03) | 0.22(.01) | 0.06(.46) | 0.07(.41) | 0.17(**.**04) |
|  | 2^nd^ ^c^ | R(*P*) | 0.09(.43) | 0.05(.65) | 0.01(.91) | 0.17(.15) | 0.03(.78) |
|  |  |  |  |  |  |  |  |
| Highest level of education | 1^st^ ^b^ | R(*P*) | 0.19(.02) | 0.21(.01) | 0.09(.26) | 0.07(.37) | 0.22(.01) |
|  | 2^nd^ ^c^ | R(*P*) | 0.20(.10) | 0.19(.11) | 0.16(.19) | 0.20(.10) | 0.16(.19) |
|  |  |  |  |  |  |  |  |
| Number of sessions attended | 1^st^ ^b^ | R(*P*) | . | . | . | . | . |
|  | 2^nd^ ^c^ | R(*P*) | 0.43(.01) | 0.38(.01) | 0.34(.01) | 0.33(.01) | 0.23(.09) |

^a^ Data was analyzed using Spearman correlation. R= correlation coefficient, *P* = significance.

^b^ Before intervention

^c^ After intervention
